# Supplementary material for: Spatial segregation of catalytic sites within Pd doped H-ZSM-5 for fatty acid hydrodeoxygenation to alkanes
Source: Nat Commun. 2024 Sep 4;15:7718. doi: 10.1038/s41467-024-51925-2 (PMC11375062; doi:10.1038/s41467-024-51925-2)
Supplement: Supplementary file 1 — Supplementary Information [file 41467_2024_51925_MOESM1_ESM.pdf]

Supplementary information

Spatial segregation of catalytic sites within Pd doped H-ZSM-5 for fatty acids hydrodeoxygenation to alkanes

Shengzhe Ding[1,2], Dario Luis Fernandez Ainaga[3], Min Hu[1], Boya Qiu[1], Ushna Khalid[1], Carmine Dágostino[1,4], Xiaoxia Ou[1,5], Ben Spencer[6,7], Xiangli Zhong[6,7], Yani Peng[1], Nicole Hondow[3], Constantinos Theodoropoulos[1], Yilai Jiao[8], Christopher M. A. Parlett[1,9,10,11*] and Xiaolei Fan[1,5,12*]

[1]Department of Chemical Engineering, The University of Manchester, Manchester, M13 9PL, UK

[2] Institute of Catalysis Science, Beijing Research Institute of Chemical Industry, Sinopec, Beijing, 100013, China

[3]School of Chemical and Process Engineering, University of Leeds, Leeds, LS2 9JT, UK

[4]Dipartimento di Ingegneria Civile, Chimica, Università di Bologna, 40131 Bologna, Italy

[5]Nottingham Ningbo China Beacons of Excellence Research and Innovation Institute, Ningbo, 315100, China

[6]Henry Royce Institute, The University of Manchester, Manchester, M13 9PL, UK

[7]Department of Materials, The University of Manchester, Manchester, M13 9PL, UK

[8]Shenyang National Laboratory for Materials Science, Chinese Academy of Sciences, Shenyang 110016, China

[9]Diamond Light Source, Harwell Science and Innovation Campus, Didcot, Oxfordshire OX11 0DE, UK

[10]University of Manchester at Harwell, Harwell Science and Innovation Campus, Didcot, Oxfordshire, OX11 0DE, UK

[11]UK Catalysis Hub, Rutherford Appleton Laboratory, Harwell, Oxfordshire, OX11 0FA, UK

[12]Institute of Wenzhou, Zhejiang University, Wenzhou 325006, China

Supplementary Equations

1. Relative crystallinity by XRD (RC)

$$RC = \frac{S_{H-MZSM5}}{S_{H-ZMS5}} \times 100$$ Supplementary Equation 1

$S_{H\text{-}MZSM5}$ = peak area of diffraction reflection in the range of 22.5–25.0° 2θ for H-MZSM5

$S_{H\text{-}ZSM5}$ = peak area of diffraction reflection in the range of 22.5–25.0° 2θ for H-ZSM5 (commercial microporous ZSM-5 post calcination).

Supplementary Figures

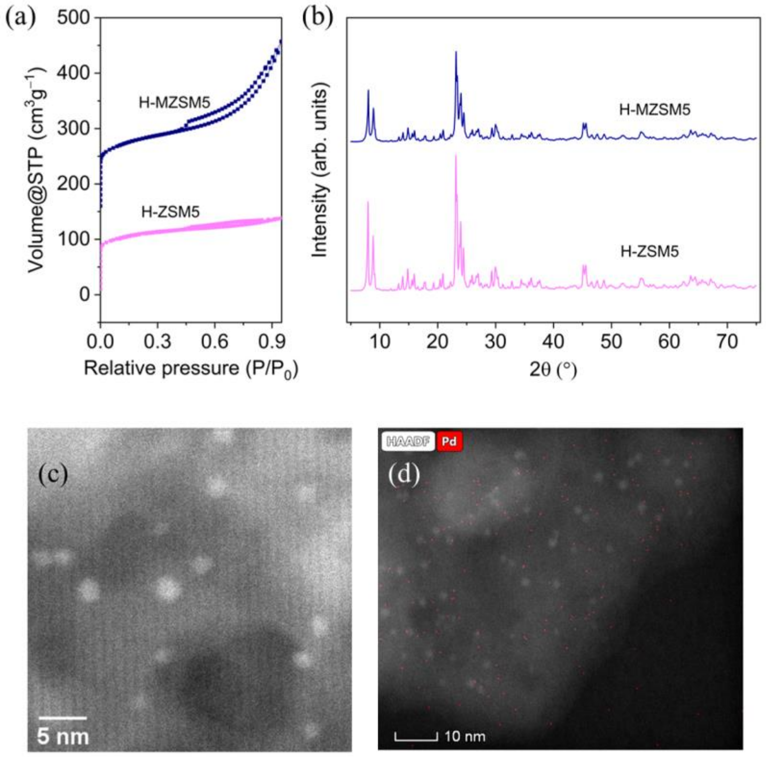

Supplementary Figure 1. (a) N_2 adsorption-desorption isotherms of H-ZSM5 and H-MZSM5; (b) wide-angle XRD patterns of H-ZSM5 and H-MZSM5; (c–d) representative HAADF-STEM images of Pd_{imp}/H-MZSM5 with EDS mapping in (d).

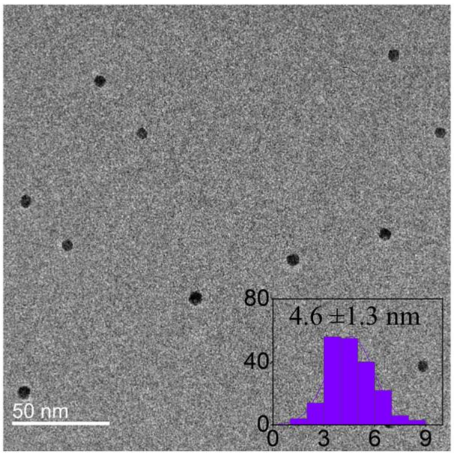

Supplementary Figure 2. Representative TEM image of the as-prepared (unsupported) Pd NPs with the associated particle size distribution in the inset. The error on the average particle sizes reported is the standard deviation of the dataset.

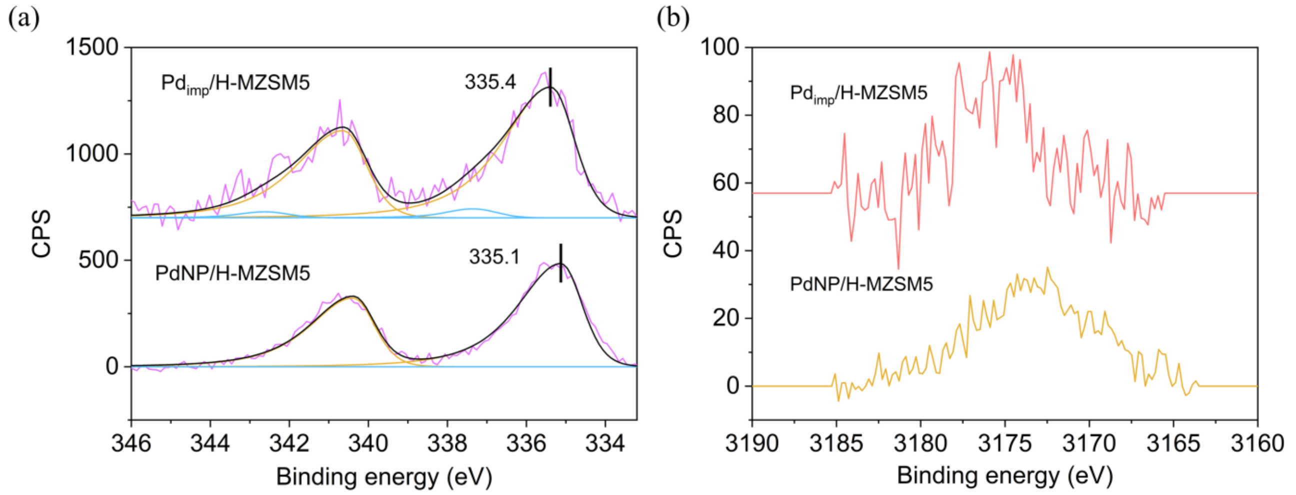

Supplementary Figure 3. Comparative XPS analysis of Pd_{imp}/H-MZSM5 and PdNP/H-MZSM5. (a) XPS profiles of Pd *3d* regions, raw data in purple, the deconvolution to Pd in yellow and PdO in blue, and the resulting fit envelope in black. The CPS of Pd_{imp}/H-MZSM5 presented in (a) are scaled by a factor of 8 to aid visualisation. (b) HAXPES profiles of Pd *2p* regions. The CPS of Pd_{imp}/H-MZSM5 presented in (b) scaled by a factor of 5 to aid visualisation.

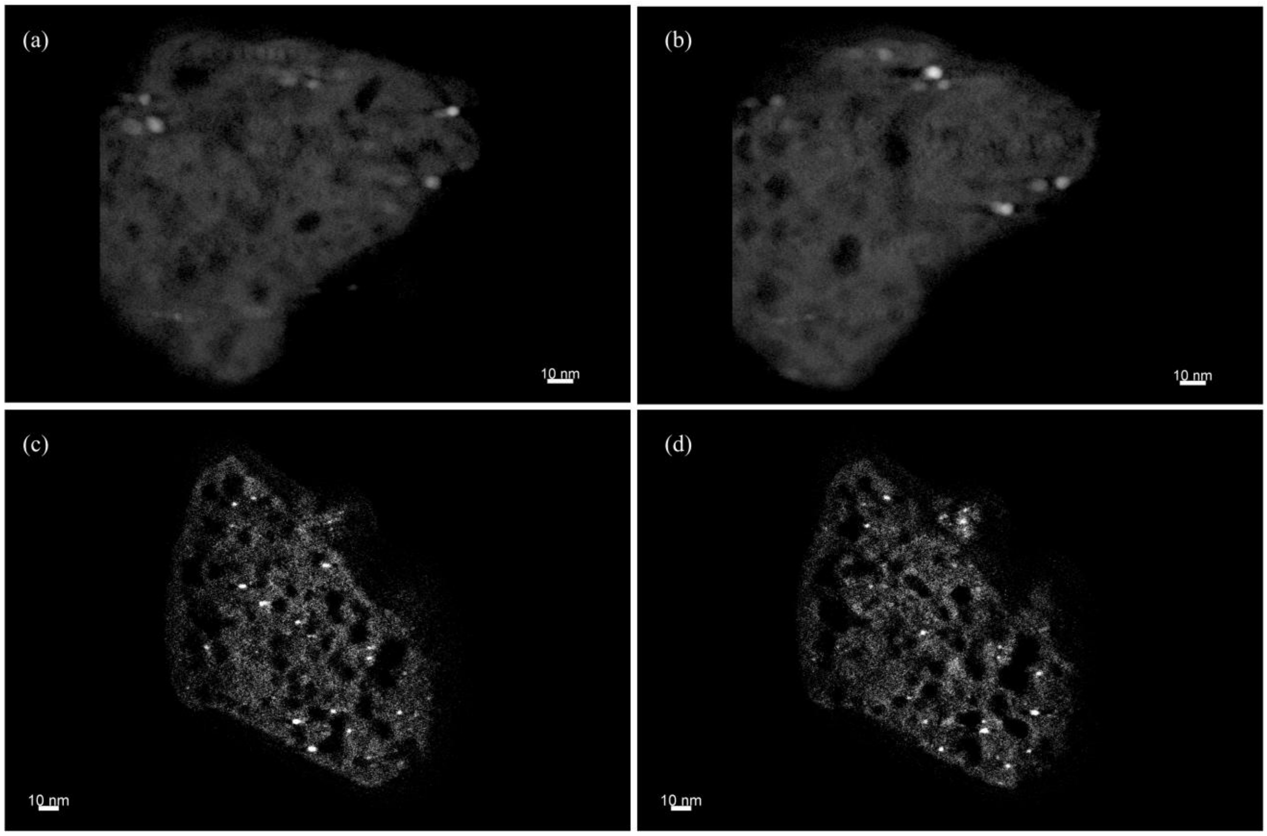

Supplementary Figure 4. The slices derive from STEM tomogram reconstructions for PdNP/H-MZSM5 (a, b) and Pd_{imp}/H-MZSM5 (c, d), with slices above (a, c) and below (b, d) those in Figure 2.

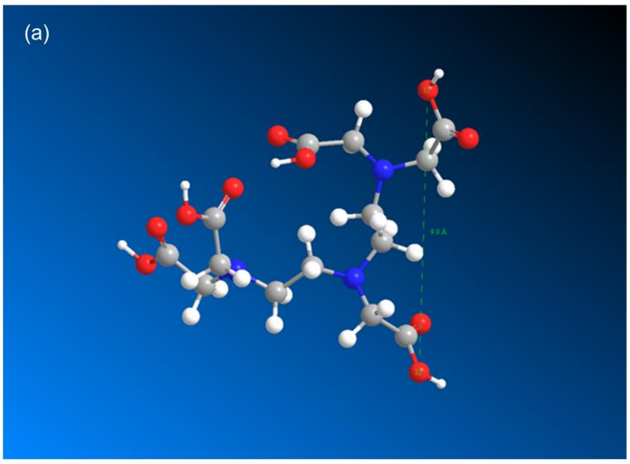

Supplementary Figure 5. Computed molecular structures of diethylenetriaminepentaacetic acid (DPTA). The geometry of (a) was calculated using the NIST Chemistry WebBook (https://webbook.nist.gov/cgi/cbook.cgi?ID=67-43-6), retrieved on March 9, 2023[1].

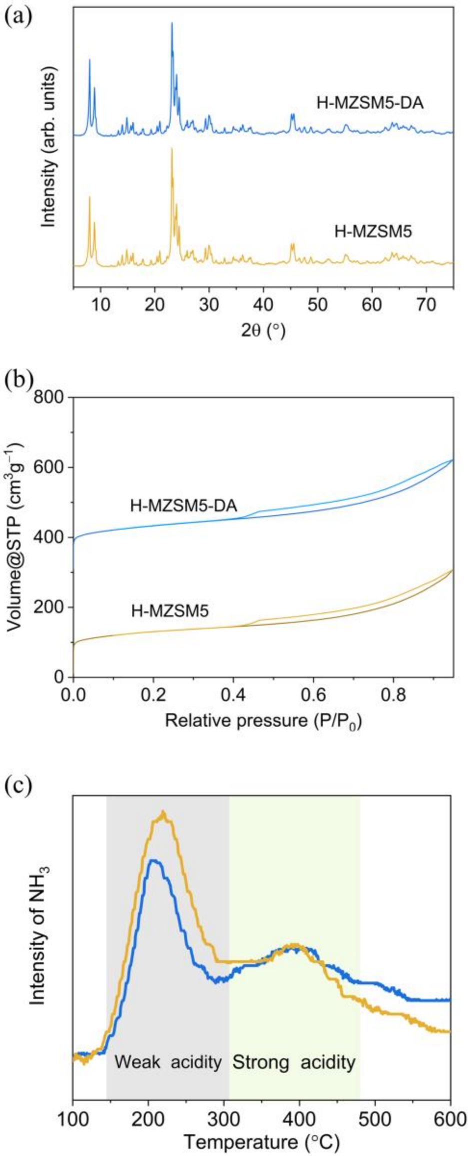

Supplementary Figure 6. Physical properties of H-MZSM5-DA. (a) Wide-angle XRD patterns; (b) N_2 adsorption-desorption isotherms of H-MZSM5 and H-MZSM5-DA; (c) mass normalised NH_3-TPD profiles of H-MZSM5 (orange line) and H-MZSM5-DA (blue line).

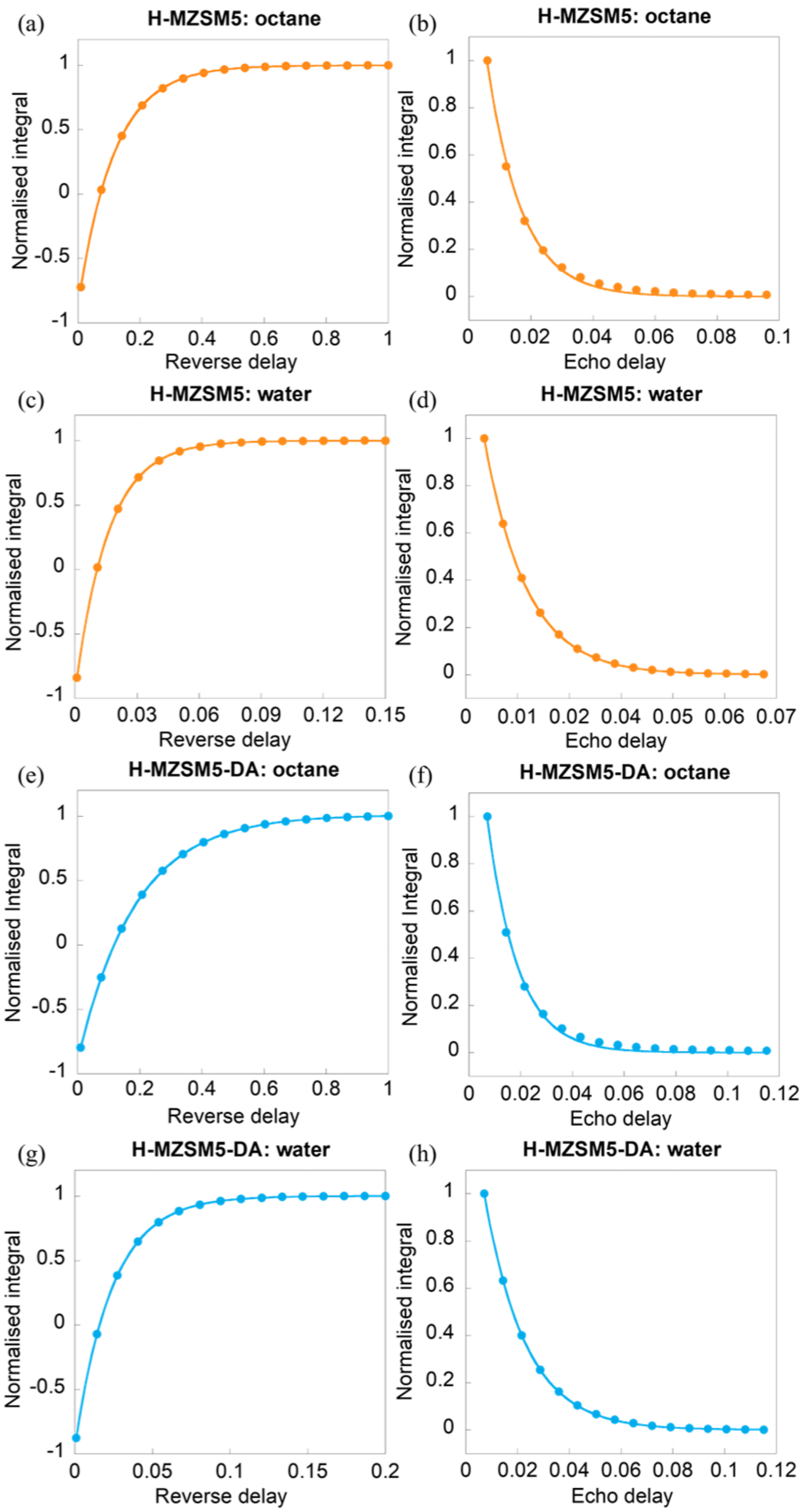

Supplementary Figure 7. T_1 and T_2 relaxation times plots of octane (a, b, e, f) and water (c, d, g, h) within H-MZSM5 (a–d) and H-MZSM5-DA (e–h) catalysts. The relative error on T_1 and T_2 are ±3%, determined from repeat analysis to assess instrument error.

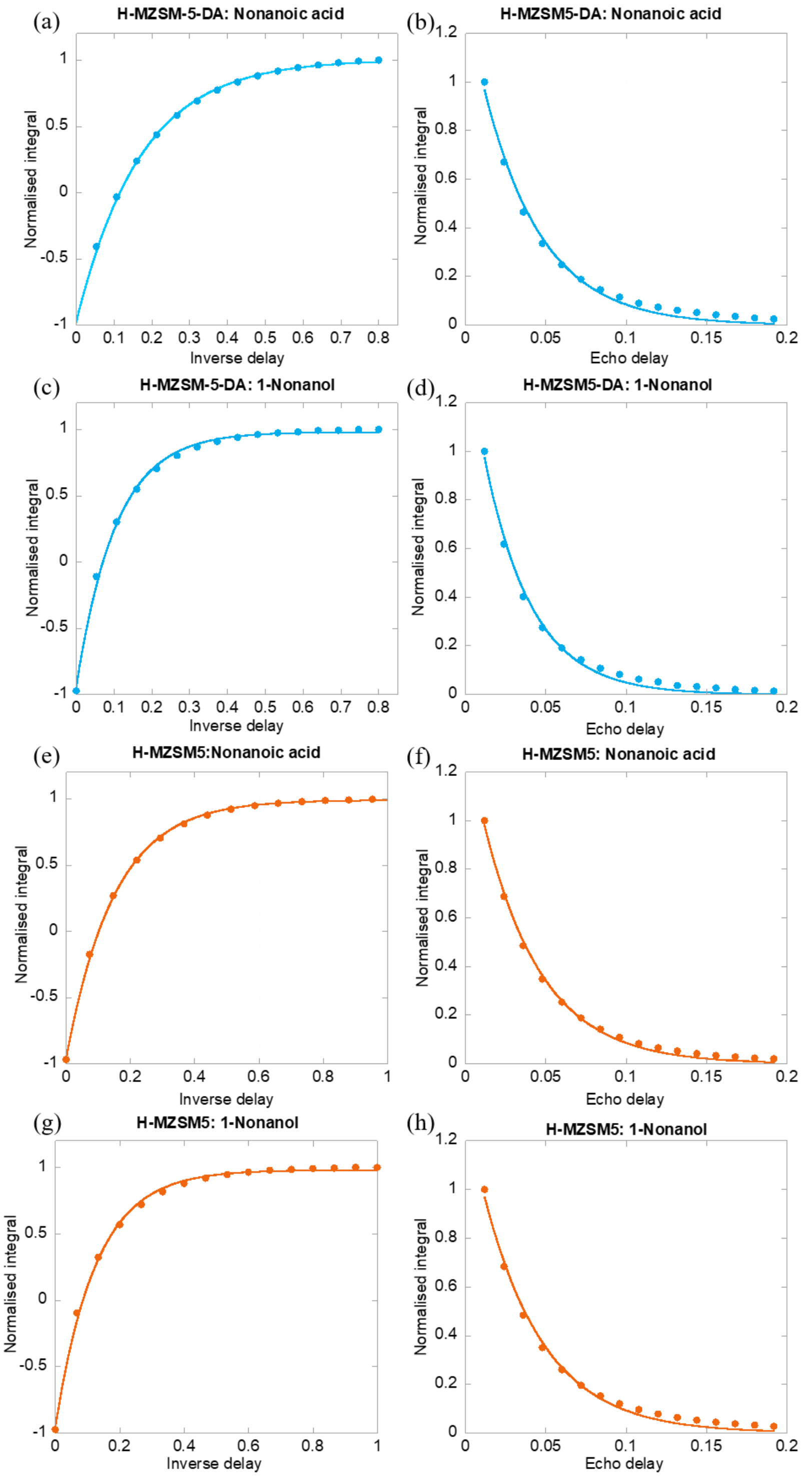

Supplementary Figure 8. T_1 and T_2 relaxation times plots of 1-nonanoic acid (a, b, e, f) and 1-nonanol (c, d, g, h) within H-MZSM5 (a–d) and H-MZSM5-DA (e–h) catalysts.

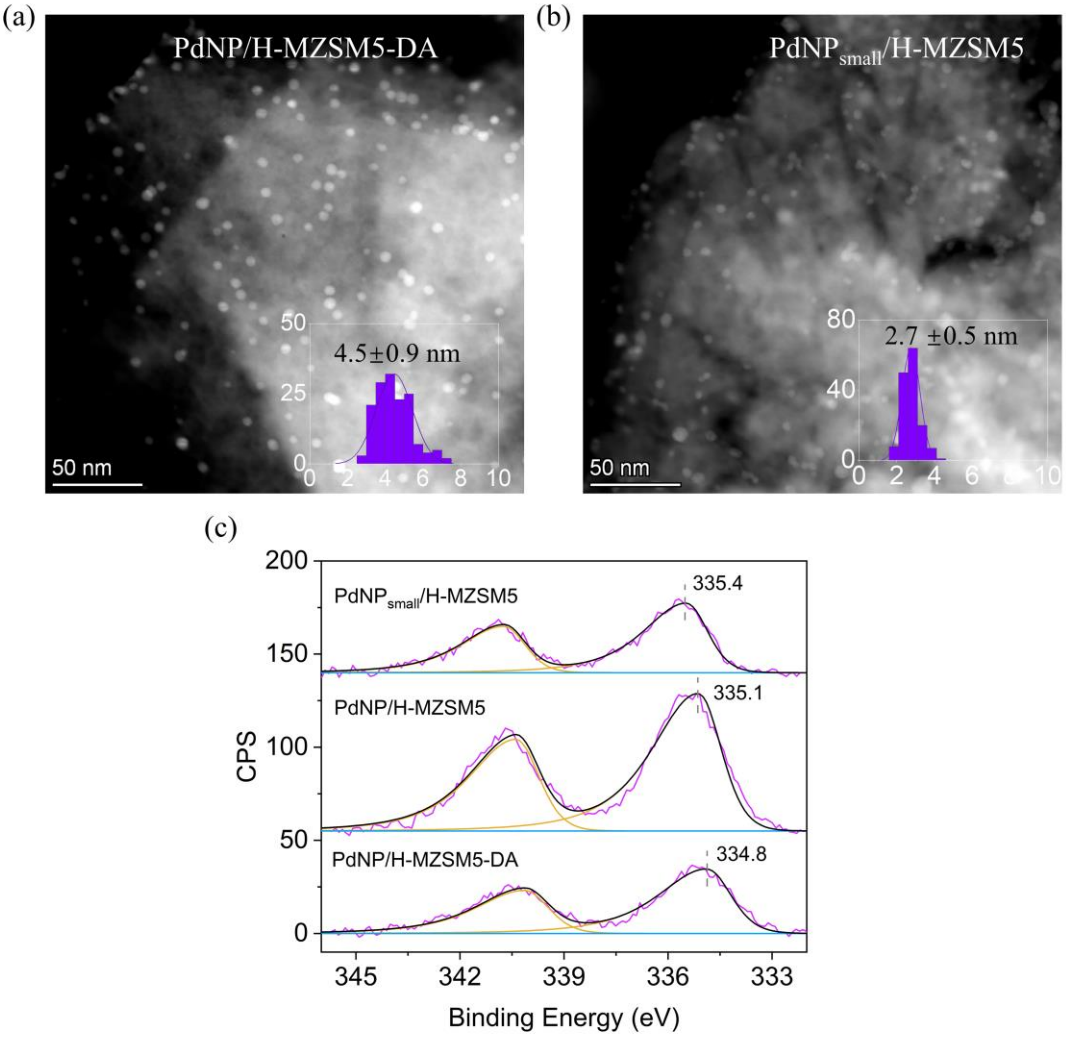

Supplementary Figure 9. (a) Representative HAADF-STEM image of PdNP/H-MZSM5-DA (with 0.82 ± 0.03wt.% Pd loading); (b) Representative HAADF-STEM image of PdNP$_{small}$/H-MZSM5 (with 0.76 ± 0.03wt.% Pd loading); (c) XPS profiles of PdNP/H-MZSM5-DA, PdNP/H-MZSM5 and PdNPsmall/H-MZSM5. The error on the average particle sizes reported is the standard deviation of the dataset.

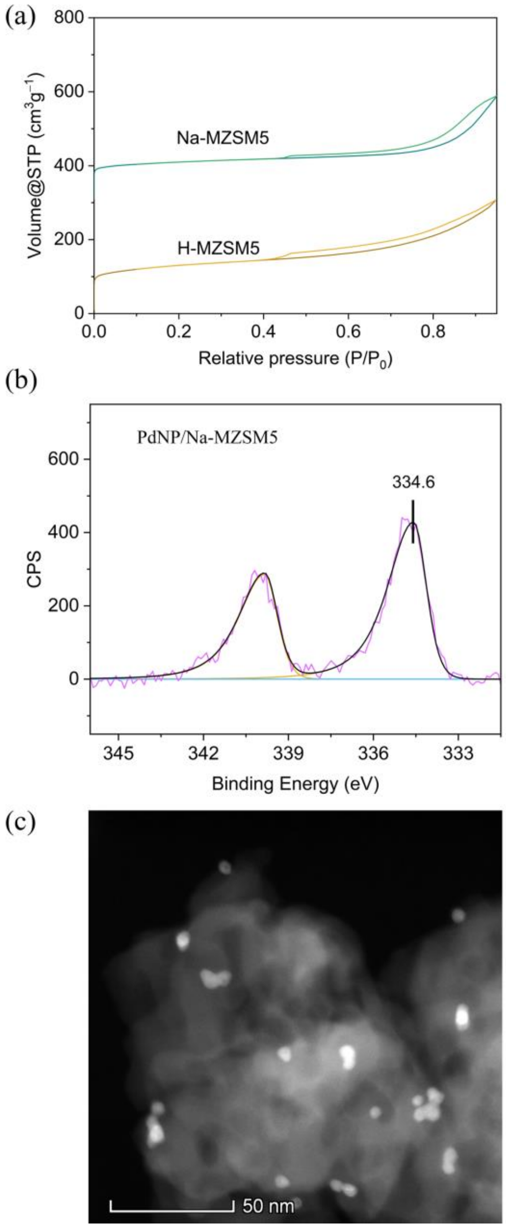

Supplementary Figure 10. (a) Nitrogen isotherm of Na-MZSM5 and H-MZSM5; (b) XPS profile of Pd *3d* region in PdNP/Na-MZSM5 (with 0.81 ± 0.03wt.% Pd loading), raw data in purple, the deconvolution to Pd in yellow and PdO in blue, and the resulting fit envelope in black; (c) representative HAADF-STEM of PdNP/Na-MZSM5.

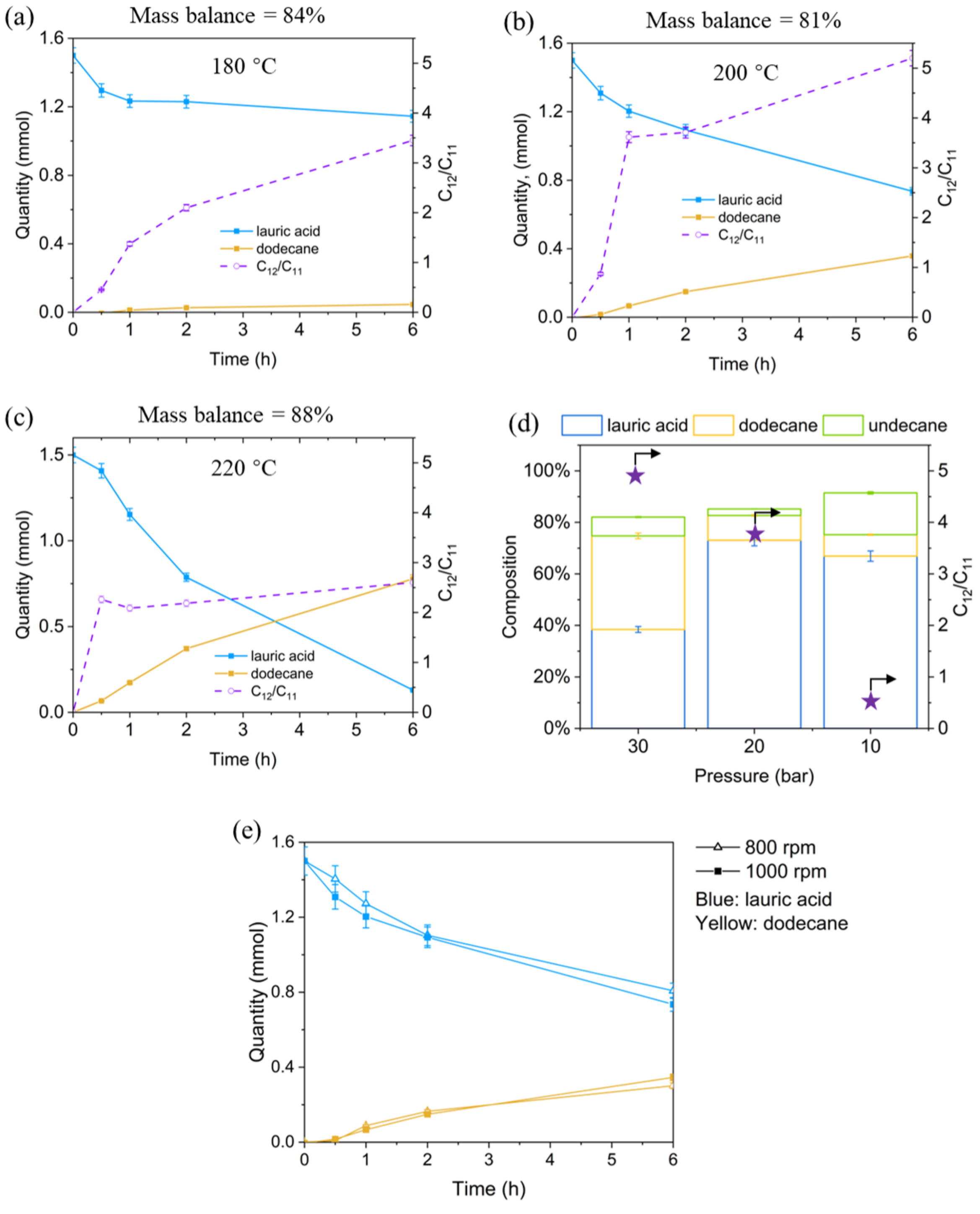

Supplementary Figure 11. Process conditions optimisation for lauric acid HDO over PdNP/H-MZSM5. Reaction profiles as a function of temperature (a) 180 °C, (b) 200 °C, (c) 220 °C; (d) product distribution of the reaction systems after the 6-h reaction under different pressures; (e) reaction profile as a function of stirring rate. The errors of conversion and selectivity are ±3% and yields are ±5%, with error bars representing the standard deviation of the average taken over at least two independent measurements. Reaction conditions: 100 mg 1 wt.% Pd-doped catalyst, 300 mg lauric acid, 0.1 cm^3 of nonane as the internal standard, 40 cm^3 of hexane as the solvent, unless stated otherwise at 200 °C, 30 bar H_2 and 1000 rpm.

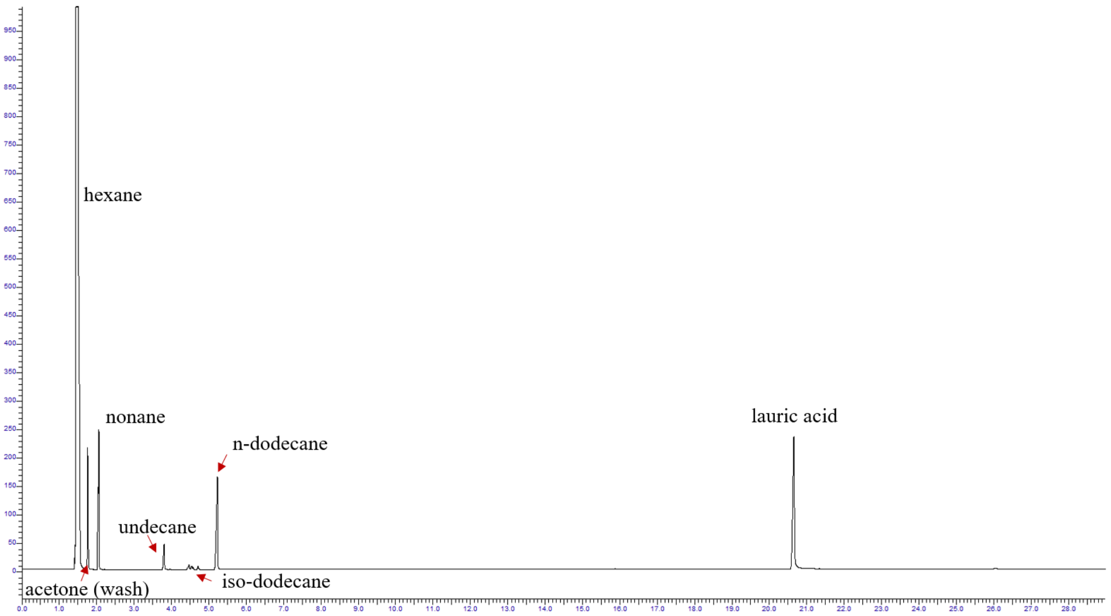

Supplementary Figure 12. Representative GC chromatogram for HDO of lauric acid over PdNP/H-MZSM5 at 200 °C, 30 bar H_2 and 1000 rpm.

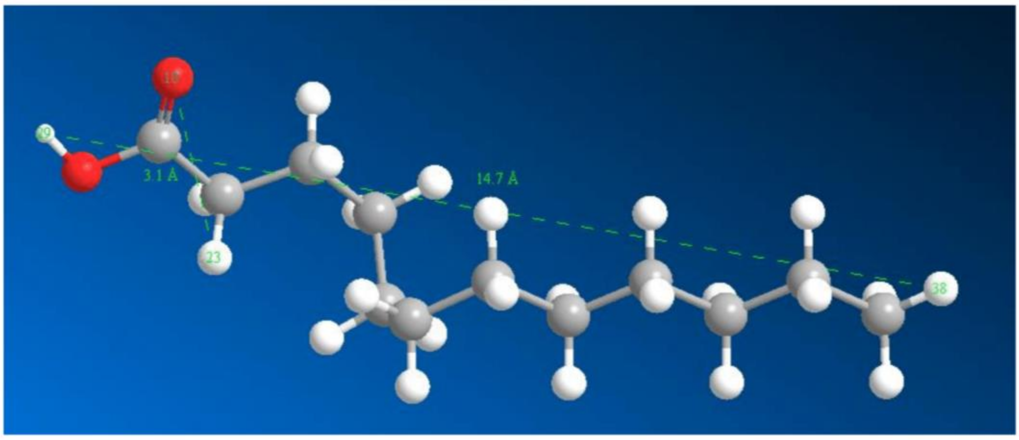

Supplementary Figure 13. Computed molecular structure of lauric acid with size estimation. The geometry of lauric acid was calculated by NIST Chemistry WebBook (https://webbook.nist.gov/cgi/cbook.cgi?Name=143-07-7&Units=SI), retrieved 09/03/2023[1].

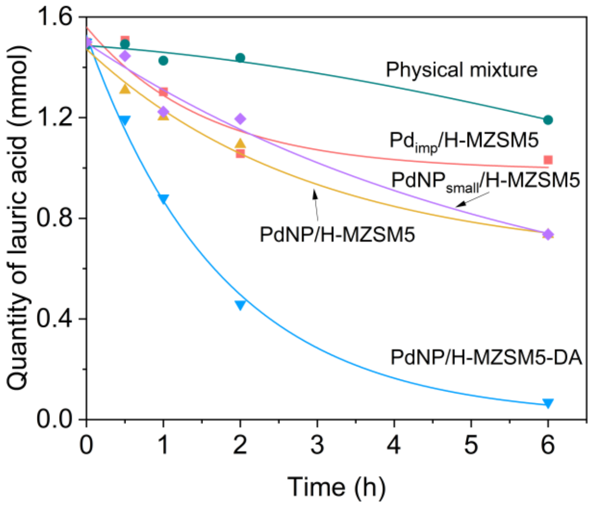

Supplementary Figure 14. Lauric acid reaction profiles for PdNP/H-MZSM5-DA, PdNP/H-MZSM5, PdNP$_{small}$/H-MZSM5, Pd$_{imp}$/H-MZSM5, and physical mixture of H-MZSM5 + PdNP/Na-MZSM5. Error bars correspond to the standard deviation of the average taken over at least two independent measurements. Reaction conditions: 100 mg 1 wt.% Pd-doped catalyst, 300 mg lauric acid, 0.1 cm^3 of nonane as the internal standard, 40 cm^3 of hexane as the solvent, at 200 °C, 30 bar and 1000 rpm.

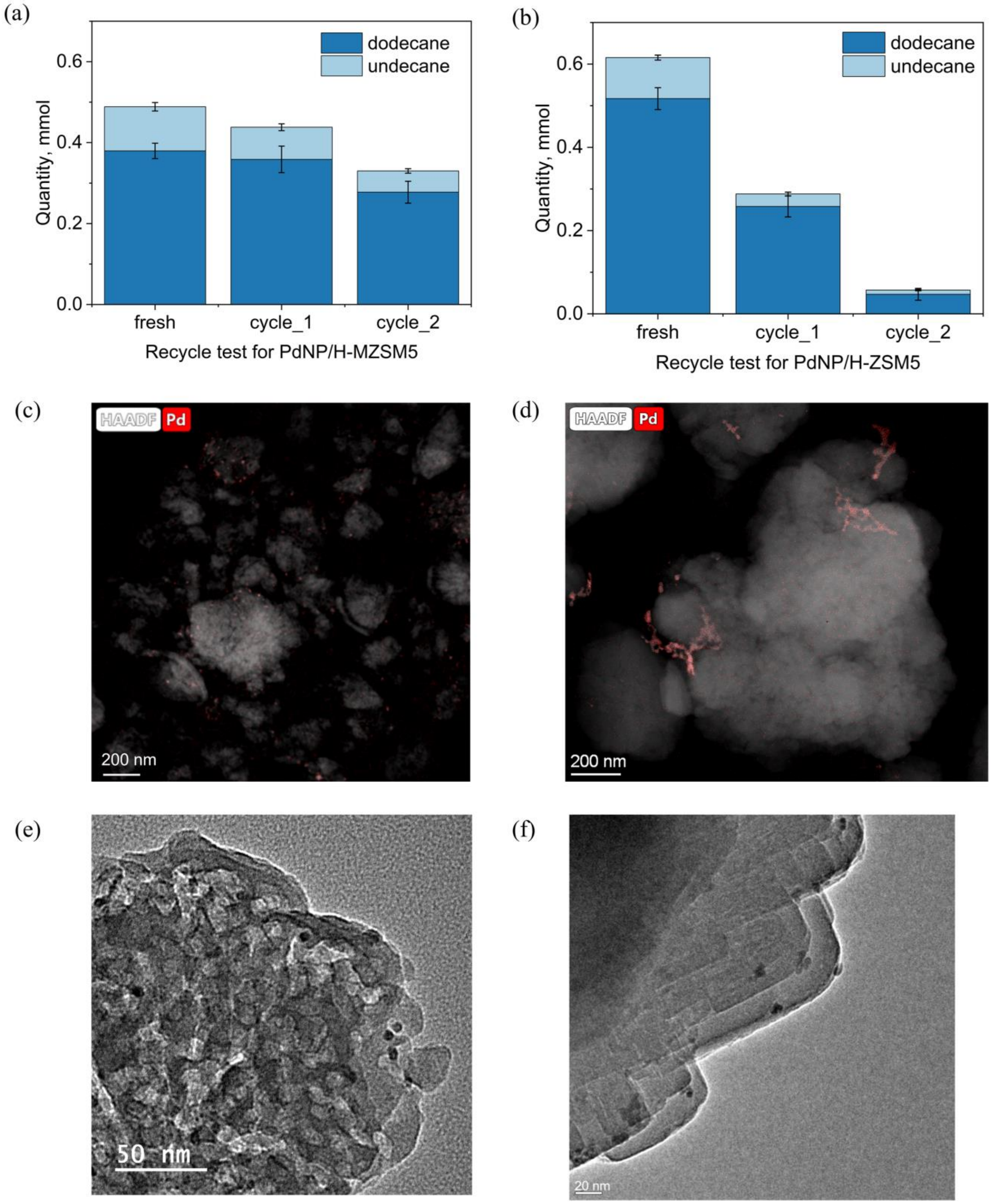

Supplementary Figure 15. Stability test for HDO of lauric acid. (a) Product yields from recycle studies for lauric acid HDO over (a) PdNP/H-MZSM5 and (b) PdNP/H-ZSM5. (c) Representative HAADF-STEM image of PdNP/H-MZSM5 after the second recycle (with EDS mapping). (d) Representative HAADF-STEM image of PdNP/H-ZSM5 after the second recycle (with EDS mapping). (e) Representative TEM image of the fresh PdNP/H-MZSM5; (f) Representative TEM image of the fresh PdNP/H-ZSM5. Error bars in (a) and (b) correspond to the standard deviation of the average taken over at least two independent measurements. Reaction conditions: 100 mg 1 wt.% Pd-doped catalyst, 300 mg lauric acid, 0.1 cm^3 of nonane as the internal standard, 40 cm^3 of hexane as the solvent, at 200 °C, 30 bar and 1000 rpm.

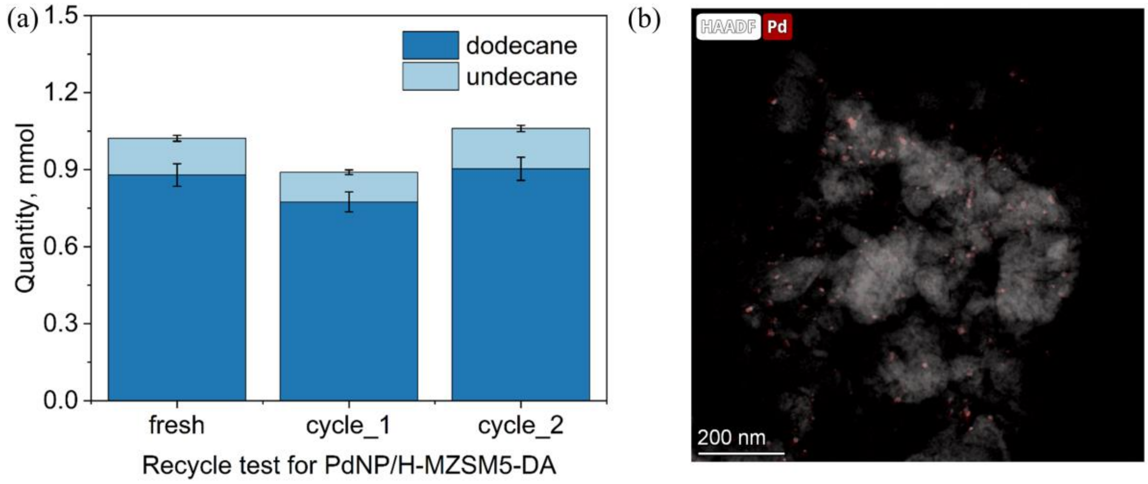

Supplementary Figure 16. (a) Product yields from recycle studies for lauric acid HDO over PdNP/H-MZSM5-DA. (b) Representative HAADF-STEM image of PdNP/H-MZSM5-DA after the second recycle (with EDX mapping). Error bars in (a) and (b) correspond to the standard deviation of the average taken over at least two independent measurements. Reaction conditions: 100 mg 1 wt.% Pd-doped catalyst, 300 mg lauric acid, 0.1 cm^3 of nonane as the internal standard, 40 cm^3 of hexane as the solvent, at 200 °C, 30 bar and 1000 rpm.

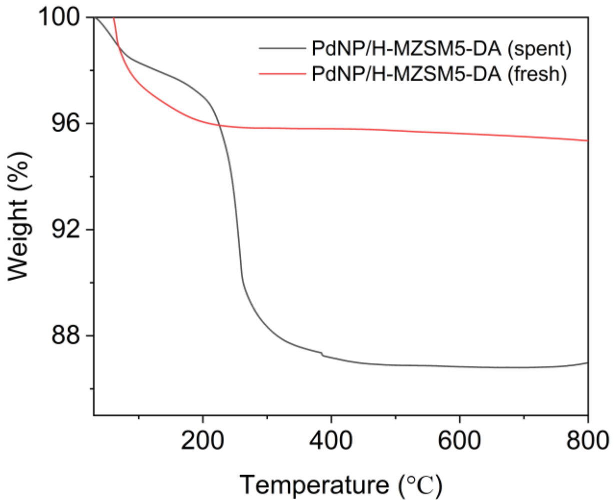

Supplementary Figure 17. Thermogravimetric analysis (TGA) of fresh and spent PdNP/H-MZSM5-DA.

Supplementary Tables

Supplementary Table 1. Porous properties of the ZSM-5 zeolites and Pd-doped zeolite catalysts.

Sample	S_{BET} ($m^2\ g^{-1}$)	S_{micro} ($m^2\ g^{-1}$)	$S_{external}$ ($m^2\ g^{-1}$)[a]	V_{micro} ($cm^3\ g^{-1}$)[b]	V_{meso} ($cm^3\ g^{-1}$)[c]	PD (nm) [d]
HZSM-5	344 ± 34	208 ± 21	136 ± 14	0.109 ± 0.01	0.061 ± 0.006	<2
H-MZSM5	419 ± 42	206 ± 21	213 ± 21	0.108 ± 0.01	0.317 ± 0.032	8.6
H-MZSM5-DA	434 ± 43	177 ± 18	257 ± 26	0.093 ± 0.01	0.342 ± 0.034	8.9
Na-MZSM5	345 ± 34	222 ± 22	123 ± 12	0.116 ± 0.01	0.304 ± 0.030	6.9
Pd_{imp}/H-MZSM5	414 ± 41	193 ± 19	221 ± 22	0.101 ± 0.01	0.328 ± 0.033	8.7
PdNP/H-MZSM5	429 ± 43	205 ± 20	224 ± 22	0.108 ± 0.01	0.298 ± 0.030	8.4
PdNP/H-MZSM5-DA	441 ± 44	215 ± 21	226 ± 23	0.130 ± 0.01	0.321 ± 0.032	8.0

[a] External surface area was determined by $S_{external} = S_{BET} - S_{micro}$; [b] determined by the t-plot method; [c] mesopore volume was determined by $V_{meso} = V_{total} - V_{micro}$; [d] PD = pore diameter, calculated from the adsorption branch using the BJH method with Fass correction. Errors are determined from repeat measurements to assess instrument error.

Supplementary Table 2. Physicochemical properties of Pd-doped H-MZSM5.

Sample	Pd loading[a] (wt.%)	Pd size[b] (nm)	Pd^{2+}/Pd^0 (-)	Pd Dispersion[c] (%)
PdNP/H-MZSM5	0.81 ± 0.03	4.4 ± 0.6	0	11.9 ± 1.2
PdNP/H-MZSM5-DA	0.82± 0.03	4.5± 0.9	0	not measured
Pd_{imp}/H-MZSM5	1.02 ± 0.01	2.2 ± 0.6	0.05	26.5 ± 2.7

[a] ICP; [b] TEM with errors based on the standard deviation of the average taken over at least 200 independent measurements; [c] calculated by CO pulse chemisorption. ICP and chemisorption errors were assessed from repeat analyses to account for measuring and instrument errors.

Supplementary Table 3. T_1, T_2, and T_1/T_2 values of different probes imbibed within the pores of the hierarchical ZSM-5.

Chemical	H-MZSM5			H-MZSM5-DA		
	T_1 (ms)	T_2 (ms)	T_1/T_2 (-)	T_1 (ms)	T_2 (ms)	T_1/T_2 (-)
Water	16 ± 0.8	9 ± 0.5	1.8 ± 0.1	24 ± 1.2	16 ± 0.8	1.5 ± 0.1
1-nonanol	126 ± 6.3	38 ± 1.9	3.3 ± 0.2	104 ± 5.2	29 ± 1.5	3.5 ± 0.2
1-nonanoic acid	150 ± 7.5	37 ± 1.9	4.1 ± 0.2	170 ± 8.5	36 ± 1.8	4.7 ± 0.2
Octane	117 ± 5.8	11 ± 0.6	10.6 ± 0.5	185 ± 9.2	12 ± 0.6	15.4 ± 0.8

Errors were assessed by repeat analysis to account for instrument error.

Supplementary Table 4. Catalytic performance of PdNP/H-MZSM5 under different conditions.[a]

T (°C)	H_2 P (bar)	Stirring rate (rpm)	Conversion (%)	n-C_{12} yield (%)	iso-C_{12} yield (%)	C_{12}/C_{11} [b] (-)
**180**	30	1000	24	6	0.6	3.5
**200**	30	1000	51	24	3	5.2
**220**	30	1000	91	52	6	2.6
**200**	10	1000	33	8	1	0.6
**200**	20	1000	27	9	1	4.4
**200**	30	1000	64	36	5	5.6
**200**	30	800	39(44)	20(19)	2(2)	4(3.9)
**200**	30	1000	51(48)	24(25)	3(3)	5.2(5.4)

[a] Reaction conditions: 100 mg PdNP/H-MZSM5, 300 mg lauric acid, 0.1 cm^3 of nonane as the internal standard, 40 cm^3 of hexane as the solvent; [b] C_{12}/C_{11} = (yield of n-C_{12} + yield of iso-C_{12}) / yield of C_{11}; data in parenthesis are the results from the repeated reactions. The errors of conversion and selectivity are within ±3%, and yields are within ±5%.

Supplementary Table 5. PdNP/H-MZSM5-DA performance for HDO of other fatty acids.[a]

**Substrate**	**Acid Conv., %**	**C_n Sel., %**	**C_{n-1} Sel., %**	**Productivity [b] (mol_{Cn} mol_{Pd}^{-1} h^{-1})**
Palmitic acid (C16)	19.2	52.4	14	13.6
Stearic acid (C18)	17.5	78.4	21.6	12.5

[a] Reaction conditions: 34 mg 1 wt.% Pd-doped catalyst, 380 mg palmitic or 420 mg stearic acid, 0.1 cm^3 of nonane as the internal standard, 40 cm^3 of hexane as the solvent, at 200 °C, 30 bar and 1000 rpm for 6 h. [b] Production rate was calculated based on the yield of C_n in 2 h. The errors of conversion and selectivity are within ±3%, whereas yields and production rates are within ±5%.

Supplementary Table 6. Lauric acid HDO performance over conventionally prepared and spatial compartmentalised catalysts.[a]

Catalysts	Acid Conv., %	C_{12} Sel., %	C_{11} Sel., %	Productivity [b] (mol_{C12} mol_{Pd}^{-1} h^{-1})
PdNP/ H-MZSM5-DA	66.7	57.4	12.5	71.0
PdNP/ H-MZSM5-DA25	73.0	50.5	9.6	53.3
PdNP/ H-MZSM5-DA15	51.7	27.9	5.6	23.3
PdNP/H-USY-DA	99.3	52.7	24.0	139.1
PdNP/H-BETA-DA	83.3	47.4	19.6	93.7

[a] Reaction conditions: 100 mg 0.4 wt.% Pd-doped catalyst, 600 mg lauric acid, 0.1 cm^3 of nonane as the internal standard, 40 cm^3 of hexane as the solvent, at 200 °C, 30 bar and 1000 rpm for 6 h. [b] Production rate was calculated based on the yield of n-C_{12} + iso-C_{12} in 2 h, as shown in Fig. 4 (a). The unit is $mol_{dodecane}$ mol_{Pd}^{-1} h^{-1}, in which the quantity of Pd is based on Pd loading from ICP. The errors of conversion and selectivity are within ±3%, whereas yields and production rates are within ±5%.

Supplementary Table 7. Comparison of different HDO catalysts.

Catalyst	Fatty acid to Pd mole ratio	Temperature and pressure	Reaction time	Conversion	HDO/DCO_x [a]	Productivity [b] ($mol_{product}\ mol_{Pd}^{-1}\ h^{-1}$)	Ref
Pd/C	140.78	300 °C, 10 bar	5 h	96%	0.05	1.3 (27.0)	2
Pd/Al_2O_3	29.86	350 °C, 14 bar	3 h	97%	0.1	0.8 (8.6)	2
Pd/Al-SBA-15	186.62	250 °C, 25 bar	3 h	65%	0.25	7.5 (37.6)	2
Pd/SAPO-31	9.54	320 °C, 20 bar	1 h	100%	0.25	1.6 (8.0)	2
$Pd@Al\text{-}SiO_2$	23.79	260 °C, 30 bar	5 h	98%	2.5	3.3 (4.7)	3
$Pd\text{-}Nb_2O_5/SiO_2$	10.43	170 °C, 25 bar	24 h	100%	20	0.4 (0.4)	4
$Pd/HPA\text{-}SiO_2$	44.46	200 °C, 30 bar	3 h	100%	13	13.8 (14.8)	5
Pd/CuZnAl + ZSM5[c]	113.37	200 °C, 20 bar	8 h	100%	100	14.0 (14.2)	6
Pd-Re/C + zeolite A	50.00	160 °C, 30 bar	6 h	87%	50	7.1 (7.3)	7
Pt/Nb_2O_5[d]	100	180 °C, 8 bar	4 h	100%	no DCOx	22 (22)	8
Pt/ZSM-5	68.66	270 °C, 65 bar	12	100%	2.48[e]	5.72	9
Pt/TiO_2	81.67	130 °C, 20 bar	8	60%	10[f]	6.13 (5.57)	10
$Pt\text{-}Re/TiO_2$	81.67[g]	130 °C, 20 bar	2.5	86%	7.1	9.36 (8.21)[g]	10
PdNP/H-MZSM5-DA	198.75	200 °C, 30 bar	6 h	94%	5.1	20.6 (24.6)[h]	This work

[a] HDO/DCO_x was calculated based on (yield of HDO products)/(yield of DCO_x products). [b] Productivity based on HDO only, with productivity based on all products reported in parentheses. In both cases, productivity is calculated based on the final conversion and selectivity reported for the corresponding reaction time, as reaction profiles are not provided in many of the literature reports. Where Pt has been used as the metal site productivity is based on these elements.[c] Pd acts to stabilise Cu, which is the proposed active species, rather than being the active species. However, given the higher cost and low earth abundance, productivity is calculated based on Pd and not Cu. [d] Solvent free conditions. [e] Based on the carboxylic acid. [f] Based on alcohol as an intermediate HDO product. [g] Calculated with only the alkane as the HDO product, i.e. full HDO. [h] Productivity is calculated at 6 h for comparison with the existing literature, i.e., at the end of the reported reaction, whereas in Table 1 in the manuscript, it is determined for optimal performance at 2 h.

References

1 Linstrom, P. J. & Mallard, W. G. NIST Chemistry WebBook, NIST standard reference database number 69, National Institute of Standards and Technology. *Gaithersburg MD* **20899** (2022).

2 Ding, S., Parlett, C. M. A. & Fan, X. Recent developments in multifunctional catalysts for fatty acid hydrodeoxygenation as a route towards biofuels. *Mol. Catal.* **523**, 111492 (2022).

3 Cao, X. *et al.* Al-modified Pd@mSiO2 core-shell catalysts for the selective hydrodeoxygenation of fatty acid esters: Influence of catalyst structure and Al atoms incorporation. *Appl. Catal. B: Environ.* **305**, 121068 (2022).

4 Shao, Y., Xia, Q., Liu, X., Lu, G. & Wang, Y. Pd/Nb2O5/SiO2 catalyst for the direct hydrodeoxygenation of biomass-related compounds to liquid alkanes under mild conditions. *ChemSusChem* **8**, 1761-1767 (2015).

5 Liu, H. *et al.* Catalytic Hydrodeoxygenation of Methyl Stearate and Microbial Lipids to Diesel-Range Alkanes over Pd/HPA-SiO2 Catalysts. *Ind. Eng. Chem. Res.* **59**, 17440-17450 (2020).

6 Guo, Z. *et al.* Highly selective conversion of natural oil to alcohols or alkanes over a Pd stabilized CuZnAl catalyst under mild conditions. *Green Chem.* **21**, 5046-5052 (2019).

7 Ullrich, J. & Breit, B. Selective Hydrogenation of Carboxylic Acids to Alcohols or Alkanes Employing a Heterogeneous Catalyst. *ACS Catal.* **8**, 785-789 (2017).

8 Kon, Kenichi, et al., Hydrodeoxygenation of fatty acids and triglycerides by Pt-loaded Nb 2 O 5 catalysts. *Catal. Sci. Technol.,* **4**, 3705-3712 (2014).

9 Murata, Kazuhisa, et al. Production of synthetic diesel by hydrotreatment of jatropha oils using Pt– Re/H-ZSM-5 catalyst. *Energ. Fuel.* **24**, 2404-2409 (2010).

10 Manyar, Haresh G., et al. Highly selective and efficient hydrogenation of carboxylic acids to alcohols using titania supported Pt catalysts. *Chem. Commun.* **46**, 6279-6281 (2010).
