# Supplementary material for: Spatial segregation of catalytic sites within Pd doped H-ZSM-5 for fatty acid hydrodeoxygenation to alkanes
Source: Nat Commun. 2024 Sep 4;15:7718. doi: 10.1038/s41467-024-51925-2 (PMC11375062; doi:10.1038/s41467-024-51925-2)
Supplement: Supplementary file 3 — Description of Additional Supplementary Information [file 41467_2024_51925_MOESM3_ESM.pdf]

Description of Additional Supplementary Files

File Name: Supplementary Video 1
Description: HAADF-STEM tomogram of PdNP/H-MZSM5

File Name: Supplementary Video 2
Description: HAADF-STEM tomogram of Pdimp/H-MZSM5.

File Name: Supplementary Video 3
Description: HAADF-STEM tomogram reconstruction of PdNP/H-MZSM5.

File Name: Supplementary Video 4
Description: HAADF-STEM tomogram reconstruction of Pdimp/H-MZSM5.
